# An AI-based approach for detecting cells and microbial byproducts in low volume scanning electron microscope images of biofilms

**DOI:** 10.3389/fmicb.2022.996400

**Published:** 2022-12-01

**Authors:** Dilanga Abeyrathna, Md Ashaduzzaman, Milind Malshe, Jawaharraj Kalimuthu, Venkataramana Gadhamshetty, Parvathi Chundi, Mahadevan Subramaniam

**Affiliations:** ^1^Department of Computer Science, University of Nebraska, Omaha, NE, United States; ^2^Civil and Environmental Engineering Department, South Dakota School of Mines & Technology, Rapid City, SD, United States

**Keywords:** microscopy images, deep learning, self-supervised learning, contrastive learning, scanning electron microscope, microbially induced corrosion, Barlow twins, momentum contrast

## Abstract

Microbially induced corrosion (MIC) of metal surfaces caused by biofilms has wide-ranging consequences. Analysis of biofilm images to understand the distribution of morphological components in images such as microbial cells, MIC byproducts, and metal surfaces non-occluded by cells can provide insights into assessing the performance of coatings and developing new strategies for corrosion prevention. We present an automated approach based on self-supervised deep learning methods to analyze Scanning Electron Microscope (SEM) images and detect cells and MIC byproducts. The proposed approach develops models that can successfully detect cells, MIC byproducts, and non-occluded surface areas in SEM images with a high degree of accuracy using a low volume of data while requiring minimal expert manual effort for annotating images. We develop deep learning network pipelines involving both contrastive (Barlow Twins) and non-contrastive (MoCoV2) self-learning methods and generate models to classify image patches containing three labels—cells, MIC byproducts, and non-occluded surface areas. Our experimental results based on a dataset containing seven grayscale SEM images show that both Barlow Twin and MoCoV2 models outperform the state-of-the-art supervised learning models achieving prediction accuracy increases of approximately 8 and 6%, respectively. The self-supervised pipelines achieved this superior performance by requiring experts to annotate only ~10% of the input data. We also conducted a qualitative assessment of the proposed approach using experts and validated the classification outputs generated by the self-supervised models. This is perhaps the first attempt toward the application of self-supervised learning to classify biofilm image components and our results show that self-supervised learning methods are highly effective for this task while minimizing the expert annotation effort.

## 1. Introduction

Deep learning algorithms have achieved impressive successes in analyzing and extracting knowledge from images through classification, object detection, semantic and instance segmentation, and other quantitative tasks (Dong et al., 2021; Zaidi et al., 2022). These methods allow domain experts in diverse fields including biology, medicine, and engineering to explore a variety of hypotheses with a high degree of confidence and with less manual effort. Deep learning approaches learn and discover latent patterns that are often impossible to specify and program for using deterministic image processing algorithms.

MIC causes wide-ranging adverse socio-economic consequences, costing billions of dollars each year (Little et al., 2020). Devising effective MIC prevention strategies requires domain experts to analyze various properties of biofilms as they grow on exposed metal surfaces. Analyzing biofilm images to understand the distribution of their morphological components including microbial cells, cell clusters, microbially induced byproducts, metal surfaces non-occluded by cells and byproducts can be highly beneficial to experts in studying the growth and evolution of biofilms. Prevention strategies for designing new coatings on metals for controlling biofilm growth can then be assessed and improved based on the distribution of these biofilm constituents on the metal surface captured in SEM images.

The overall objective of this work is to develop automated image analyses and knowledge extraction methods to detect the distribution of various biofilm constituents over different regions of an exposed metal surface. In order to detect the presence of biofilm constituents across different regions of a biofilm image, the image is first divided into smaller image patches and deep learning classifiers are trained to detect the presence of each constituent (class object) in each patch. This information is used to automatically derive the distribution of these class objects in each image. Manually detecting these class objects and determining their distributions in different regions is a tedious and time-consuming process. Images lacking sufficient resolution make it difficult for experts to manually detect certain class objects in such images with certainty. Tools such as BiofilmQ (Hartmann et al., 2021), ImageJ (Rueden et al., 2017) that have been used for microscopy image analyses are not effective in analyzing biofilm images where the class objects appear in bursts of high density and separating cells from clusters and byproducts is often ambiguous (Ragi et al., 2021). On the other hand, deep learning methods can be used to build models that can accurately and automatically detect each of the above three class objects in each image patch and these results can then be used to automatically build heatmaps depicting the distribution of these class objects in each image.

However, building high-performing deep learning models for detecting these class objects using SEM biofilm images is a non-trivial problem. For deep learning methods require a large volume of expert-labeled images to learn patterns underlying these class objects that they can subsequently use to detect these objects. Generating large volumes of images is problematic since developing biofilms typically takes several days and is usually performed in batches of limited sizes. In addition, due to the crowded nature of these films, accurately annotating each image is labor intensive. Further, SEM images are captured under different resolutions using different magnification scales leading to cells objects with varying shapes and sizes and this makes it difficult to learn their features, especially with a small amount of data. Our approach utilizes the state-of-the-art self-supervised learning methods at the image patch level along with image processing and other pre-defined deep learning models in order to address the above challenges and build high performing models for biofilm image analyses. Biofilm images are pre-processed using contrast level enhancement based on histogram equalization (CLAHE) to sharpen the class objects in each image (Lam et al., 2017). We then apply pre-defined super-resolution deep learning models to improve the resolution of class objects and reduce the size and shape variations introduced while capturing SEM images. The available data volume is amplified by dividing each image into a large number of patches which are used to train the deep learning models. Self-supervised learning approaches first learn the representations underlying these unlabeled patches without any expert involvement. These representations are then fine-tuned to detect class objects in the image patches using a very small (less than 10%) expert annotated patches.

We have applied the proposed approach to automatically analyze SEM images of biofilms involving sulfate-reducing bacteria (SRB) grown on mild carbon steel and detect the presence and distribution of three-class objects—cells (and cell clusters), microbial byproducts, and non-occluded surface—across different regions of a biofilm SEM image. We have conducted multiple experiments to evaluate, (1) the applicability and effectiveness of the usage of state-of-the-art self-supervised techniques, (2) compare and contrast the two major self-supervised categories (contrastive and non-contrastive), (3) performance comparison between self-supervised approaches and their fully supervised counterparts on the biofilm dataset, and (4) qualitative analysis of self-supervised model performance through expert feedback.

Object classification is one of the fundamental tasks in machine learning. Over the years, image classification has been used extensively in many major domains in their applications, such as medical, engineering, and material science. Previous attempts mostly consist of pipelines with hand-crafted feature extraction to feed into the classification models. Several image processing techniques such as morphological techniques, Fourier spectrum and Wavelet coefficients, and texture and clustering have been used to extract useful feature representations (Soda and Iannello, 2009). Scale Invariant Feature Transform (SIFT) (Lowe, 2004), Speeded Up Robust Features (SURF) (Bay et al., 2006), Hough transforms (Hough, 1962), and Geometric hashing (Mian et al., 2006) are some of the popular traditional computer vision techniques to extract features for image classifiers.

Deep learning approaches have significantly outperformed the hand-crafted feature extraction approaches and are currently the defacto approach for object classification tasks. These approaches are primarily based on the supervised machine learning methods which generate models that can automatically detect objects in images by training them on images annotated by experts. Due to the large volume of expert annotated images needed to train these models several alternative machine learning methods that work with scant annotated data are being developed (Tajbakhsh et al., 2020; Chakravarthy et al., 2022), Self-supervised learning is a popular method categorized under the techniques supported by scant annotations. In self-supervised learning, first representations are learned from input data without any expert annotations and then these learned representations are fine-tuned to perform the downstream object classification task using scant expert annotations. Currently, there are mainly two types of self-supervised learning approaches, namely contrastive and non-contrastive. Some of the popular frameworks proposed in the category of contrastive self-supervised learning are SimCLR (Chen et al., 2020a) and MoCoV2 (He et al., 2020) which use both positive and negative pairs to learn representations from unlabeled data. Non-contrastive frameworks such as SimSiam (Chen and He, 2020), BYOL (Grill et al., 2020), and Barlow Twins (Zbontar et al., 2021) have shown a higher capacity to learn powerful representations only considering the positive pairs (Hence no contrasting negative pairs). Further, compared to contrastive learning, the non-contrastive learning frameworks require smaller sized batches to train the models (Tian et al., 2021). In the literature, there are several applications in a variety of domains that have been carried out with both contrastive and non-contrastive approaches (Jamaludin et al., 2017; Tajbakhsh et al., 2019; Azizi et al., 2021; Bommanapally et al., 2021).

In this paper, we focused on the self-supervised learning method for the biofilm image classification task since the annotation process of these SEM images requires significant domain expertise and is extremely time-consuming. Bommanapally et al. (2021) proposed self-supervised learning based on SimSiam framework to analyze SEM biofilm images to detect the images containing microbial byproducts. They use expert annotated images for representation learning as well as the downstream task, which makes it different from self-supervised learning methods. Further, requiring experts to annotate all images and detecting only byproducts without distinguishing them from other classes, makes this approach impractical in practice. The proposed approach uses a limited number of expert annotated images for the downstream task only in addition to performing a comprehensive classification of cells and non-occluded areas besides MIC byproducts. Ragi et al. (2021) developed deep learning-based image segmentation approaches to automating the extraction of quantitative parameters of cells in biofilms. Ragi et al. (2021) developed two deep learning-based image segmentation approaches to automate the extraction of quantitative parameters of cells in biofilms. This work employs supervised learning methods and focuses on image segmentation task and is very different from the approach described in this paper. In this regard, to the best of our knowledge, this is the first attempt to apply self-supervised learning for a comprehensive classification of SEM biofilm image components.

## 2. Materials and methods

**Figure 1:**
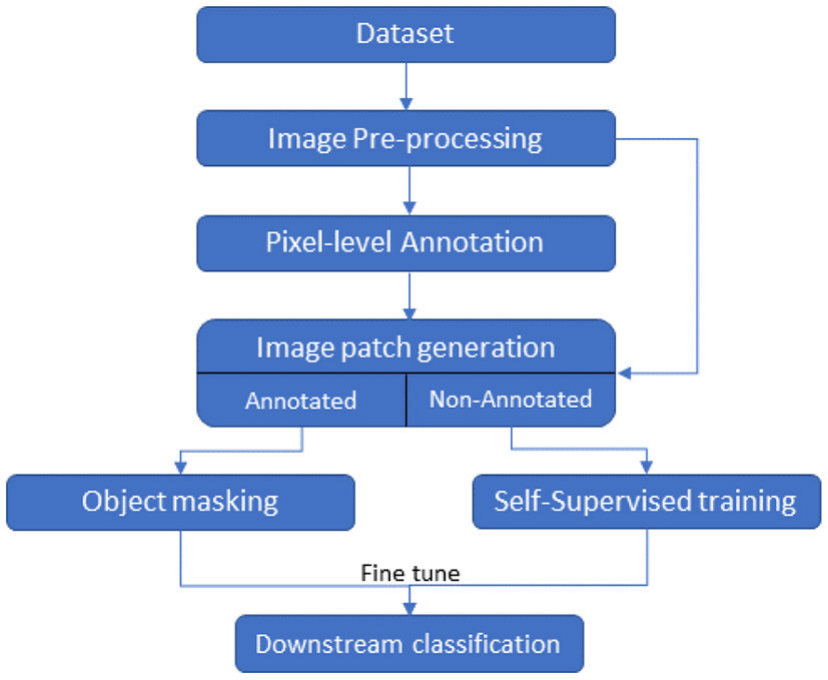
Flow chart of the classification task.

In this section, we describe our deep learning pipeline based on contrastive and non-contrastive self-supervised approaches to classify biofilm images, the dataset, the self-supervised approaches, and the evaluation metrics to assess the performance of the models. The pipeline with components is depicted in Figure 1. The pipeline takes a set of SEM raw images as input and generates a pre-processed image corresponding to each input image. The pre-processed images are manually annotated by experts. All the pre-processed images (annotated and non-annotated) are divided into patches. Here, a patch is defined as a size *m* × *m* square-shaped region of an image size *N* × *M*, where *m* << *N* and *m* << *M*. The non-annotated image patches are used to learn representations using self-supervised approaches whereas annotated patches are used to fine-tune the models for the downstream classification task. We describe the details of different components of the pipeline in detail below.

### 2.1. Dataset

While the pipeline applies to image classification in general, our focus in this paper is on the classification of Scanning Electron Microscope (SEM) images. This dataset consists of a set of SEM images of Desulfovibrio alaskensis (DA-G20), a sulfate-reducing bacteria (SRB) and their biofilms on bare mild steel surfaces grown in MIC experiments conducted in batch. Owing to its high ductility, weldability, and low cost, mild steel remains a popular choice of metal in civil infrastructure, transportation, oil and gas, and other industrial applications. However, under aqueous conditions, mild steel is susceptible to MIC caused by microorganisms including the SRB. DA-G20 was cultivated and grown in lactate C media with the following media components including sodium lactate (6.8 g/L), sodium citrate (0.3 g/L), sodium sulfate (4.5 g/L), ammonium chloride (1 g/L), dehydrated calcium chloride (0.06 g/L), potassium phosphate monobasic (0.5 g/L), magnesium sulfate (2 g/L), and yeast extract (1 g/L). The culture medium was deoxygenated by filter-sterilized nitrogen gas purging for 20 min at 15 psi followed by sterilization at 121°C for 20 min at 15 psi. The pure cultures of DA-G20 were inoculated in 150 mL serum bottles containing 80 mL of lactate-C medium and were incubated at 30°C and 125 rpm in an orbital shaker. The exponential phase pure cultures (10%) of DA-G20 were prepared and inoculated (40 mL) into the Paracell Kit (Gamry Instruments) using low carbon steel electrodes with the volume of 360 mL lactate C media. All the electrochemical measurements and their results were discussed in our recent publication (Susarla et al., 2021).

After 30 days of incubation, the biofilm surface morphology on the low carbon steel electrodes was analyzed using SEM. The biofilm samples were gently rinsed with double distilled water followed by fixation using glutaraldehyde (3%) in sodium cacodylate buffer (0.1 M, pH 7.2) (Dedysh et al., 2015). The samples were then washed subsequently using 25, 50, 75, and 100% acetone and dried overnight in a desiccator. The dried samples were then sputter-coated with a gold film and analyzed using Zeiss Supra40 variable pressure field-emission SEM fitted with an Oxford Aztec Energy advanced system. The SEM was controlled using an accelerated voltage of 5 kV.A set of these images along with meta-information such as magnification level, scale, time and date, etc. The dataset used in this paper consists of 7 grayscale SEM images at a resolution of 1024 × 758 with magnifications ranging from 436X- 2.30KX for the scale ranges from 2 to 10 micrometers.

### 2.2. Image pre-processing

Image pre-processing is an important step in computer vision tasks to improve the quality of the images for a particular application. Better performance can be achieved by cleaning the image data to the desired quality and format. Each image in the dataset includes meta-information in a black strip such as magnification level, scale, time and date, etc. This meta-information was removed in each image by manually cropping out the black strip and then, contrast enhancement (Lam et al., 2017) was applied to improve the clarity of the images. Often, the SEM images are captured by domain scientists at different scales of magnification resulting in object sizes. This is especially problematic for low volume datasets where it is common to divide an image into patches (see below for more details) to augment data volumes. Objects with vastly varying sizes make it challenging to produce image patches of a single size where objects are contained in the patch. In order to enhance image details and normalize object sizes across different magnification scales, image resolution enhancement techniques were applied to SEM images with higher micrometer scale images and lower magnification. The images with relatively lower scale and higher magnification were not undergone any resolution enhancement process.

Different image resolution enhancement techniques, specifically super-resolution techniques such as BSRGAN (Zhang et al., 2021), Real-ESRGAN (Wang et al., 2021), and SwinIR (Liang et al., 2021) using different magnification scales were applied to the raw images to produce multiple high-resolution image samples at different magnification scales (2X and 4X). These samples were analyzed using a blind voting approach by experts to select the best sample. Four experts analyzed each of these samples and voted along with justifications on the best samples. Voting ties among the experts and samples were arbitrarily broken. Based on this methodology, the BSRGAN super-resolution technique with 4X scaling was chosen for improving the resolution and clarity of the images in the dataset. After applying the BSRGAN super-resolution technique to the images, high-resolution images were obtained from the original biofilm dataset, and each high-resolution image had a resolution of 4096×2728.

### 2.3. Annotation, patch generation, object masking

Machine learning approaches typically require experts to annotate images by assigning class labels to images and their components. The expert-labeled images serve as ground truth those are used by the algorithms to learn and to test the performance of these learning algorithms. In the current work, three classes—(i) *Byproduct*, (ii) *Cell*, and (iii) *Surface*—were identified and labeled by the experts in each image in the dataset. Cells were marked in blue color, byproducts in pink, and surface in green. Image annotations were performed using the image labeler package in Matlab.

The authors who are biofilm and microscopy experts labeled the cells based on the unique features including cell shape, structure, and size of the bacterium used. However, byproducts include complex substances such as biofilm microstructure, corrosion products, and microbial products that do not have unique shapes and structures. These domain experts categorized these structures all into the same category called byproduct. Future studies are warranted such as surface elemental analysis combined with microscopic techniques to specifically categorize these structures.

Note that the problem addressed in this paper is a multi-label classification task, i.e., whether an SEM image patch belongs to more than one class we did not differentiate between cells and cell clusters in this work. Although pixel-level annotations are typically used for semantic segmentation tasks and bounding boxes are used for object detection tasks, our paper uses pixel-level annotations for effective the multi-label classification of SEM images. We also did not differentiate between pristine and contaminated surfaces in this work. Our initial analyses of the data indicated that cells and cell clusters co-occur in all images and hence cells and cell clusters are equivalent for classification. Similarly, all surface patches in the images were found to have byproduct particles and hence pristine and contaminated surface were treated equivalent for classification. However, the proposed approach can be easily extended to consider these additional classes by extending the annotations and incorporating pristine surface data samples. Though all the images in the dataset were annotated, only a small portion (~10%) of the annotated images were used by the self-supervised learning methods for classification.

**Figure 2:**
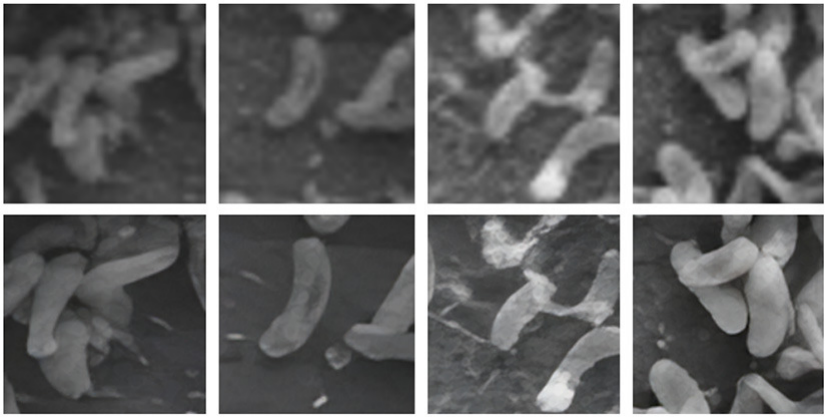
Output patches after super-resolution method. **Top** row contain patches from original image patches. **Bottom** row contains patches after applying super-resolution.

**Figure 3:**
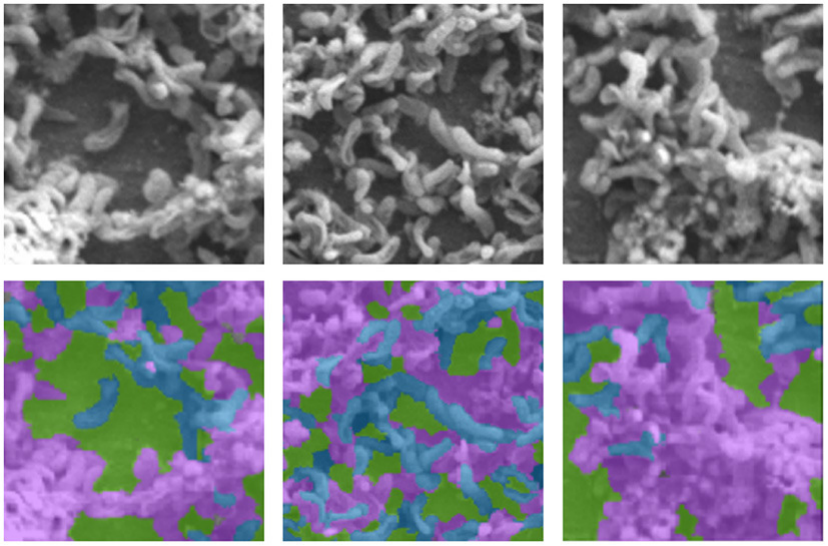
Sample SEM images (first row) with their respective annotations (second row), where *Byproduct, Cells*, and *Surface* indicates by the colors pink, blue, and green, respectively.

In general, large volumes of data are required to train high performance deep neural networks (Huang et al., 2019). However, it is challenging to produce biofilm images in high volumes as the growth of each individual film is grown in limited size batches and takes several hours (days) and batch sizes. In order to amplify the data volume available for training the models, each image was decomposed into multiple patches. We have used the traditional sliding windows technique (Ciga et al., 2021; Tsai et al., 2022) to split an image into smaller patches using different kernel sizes and different overlapping ratios (strides). The kernel size defines the dimensions of the patch and the stride gives the next region of the image from which the patch is extracted. Overlapped patches were used to reduce feature loss due to sharp patch boundaries. Four high-resolution image patches and their counterparts cropped using the same image coordinates from raw images are depicted in Figure 2. Figure 3 illustrates raw image patches and their corresponding ground truth annotations, cropped from the same image coordinates.

**Figure 4:**
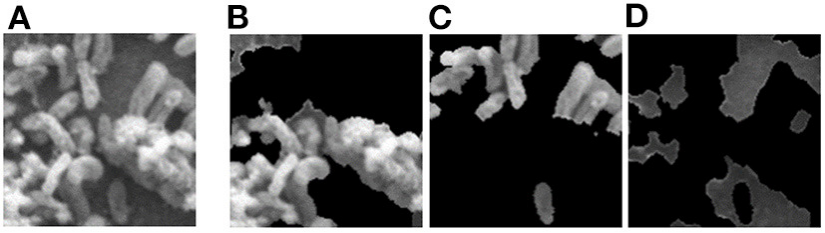
Object masking example. **(A)** Original image patch, and its object masks of **(B)** *Byproduct*, **(C)** *Cell*, and **(D)** *Surface*.

Object masking technique renders the foreground image in such a way that the target object is visible clearly and other objects invisible or less visible (Li et al., 2004). It is a powerful technique, not only to isolate the target objects but also to maintain the spatial distribution of the objects in the image. The object masks are generated from each image patch using the pixel-level annotations (pink, blue, and green), for the target objects *Byproduct, Cell*, and *Surface*, respectively (depicted in Figure 4). The masked objects appear in black in the masked patch whereas the target object instances appear in gray in the masked patch. The variation in the intensities of the target object in the masked patches depends on their intensities in the patch input for masking. For a patch that contains objects with multiple classes, multiple masked patches with pixel-level information about each of the three target objects are generated. Then based on the three target objects in these masked patches, an image-level binary label is assigned to the these masked patches and used to conduct multi-label image classification task. These masked patches with image-level annotations are then be used as the ground truth for multi-label classification task, for both training and evaluation purposes (see below for details). Note that, from this point, the masked patches with pixel-level information is no longer used.

### 2.4. Self-supervised learning

Our approach is designed to incorporate both contrastive and non-contrastive self-supervised learning algorithms to compare their efficiency on SEM biofilm images. Based on popularity, efficiency, and consistency with smaller training batches, we chose the contrastive self-supervised framework MoCoV2 (He et al., 2020) and the non-contrastive framework Barlow Twins (Zbontar et al., 2021) for learning representations from the image patches and performing the downstream classification task. Both these methods use their own mechanisms to extract complete representations consuming smaller batch sizes, which suits better with the approach discusses in this study. However, the approach is adaptable to other contrastive and non-contrastive self-supervised learning methods as well.

#### 2.4.1. MoCo: Momentum contrast for unsupervised visual representation learning

**Figure 5:**
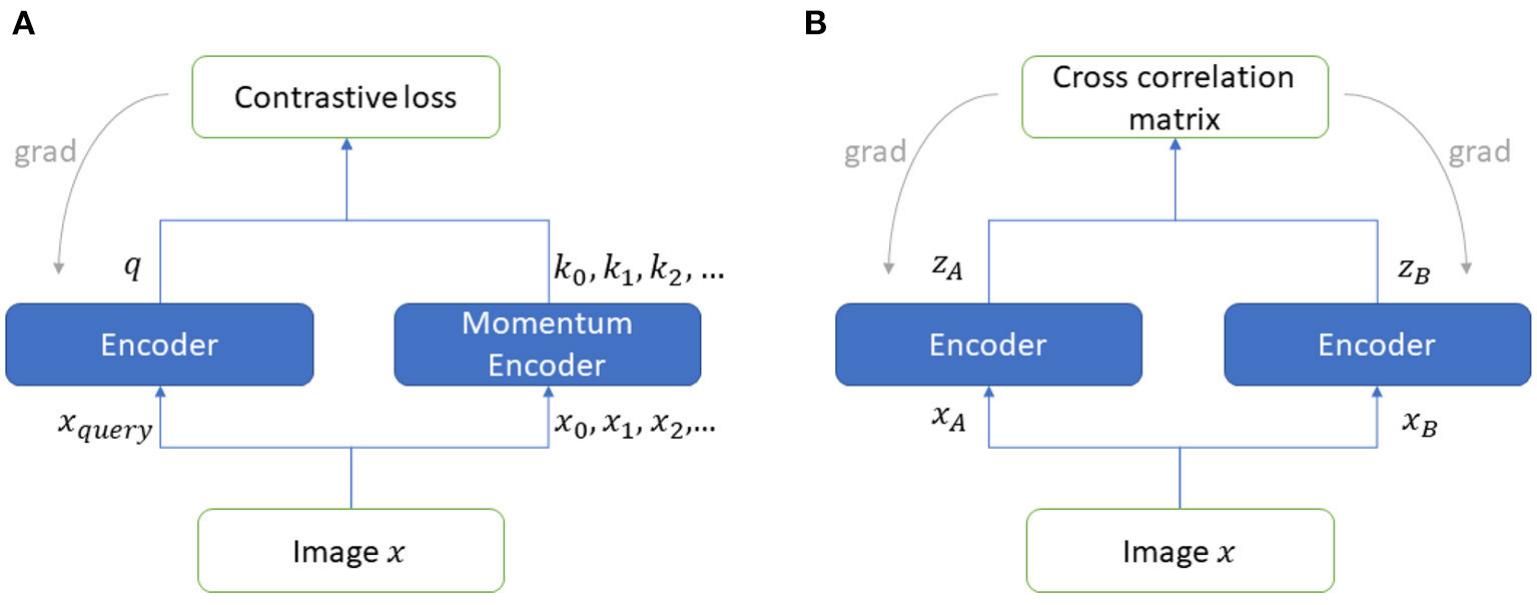
Self-supervised frameworks **(A)** MoCoV2, **(B)** Barlow Twins.

The contrastive learning approach assumes that a dataset has images where some images belong to the *positive* class and the rest belong to the *negative* class. The model learns the similarity of the positive pair of images and dissimilarities from the negative pairs of images. In MoCoV2, two encoders namely query encoder and momentum encoder take two (differently) augmented versions of the same unlabeled image and generate representations. Here, the input image is called query (*q*) whereas the encoded representations called keys (*k*^+^ represents the positive key sample matches with *q*, *k*^−^ represents the negative samples mismatch with *q*). The momentum encoder maintains a queue of keys for contrastive loss (InfoNCE) calculation purposes (see Equation 1 below). Here, the temperature parameter(τ) scales the similarity scores. MoCoV2 keeps the size of the mini-batch very small and stores the results with higher memory size. The framework for MoCoV2 is shown in Figure 5A.


(1) $$\begin{array}{l} infoNCE_{\left(q\right)}=-log\frac{exp\left(q.k^{+}/\tau \right)}{exp\left(q.k^{+}/\tau \right)+\sum_{k^{-}}exp\left(q.k^{-}/\tau \right)} \end{array}$$

#### 2.4.2. Barlow twins

Barlow Twins' self-supervised framework (Zbontar et al., 2021) utilizes two encoder models to learn representations taking unlabeled augmented data as the input. This framework is inspired by the redundancy reduction principle (efficient coding hypothesis) in the work of neuroscientist Horace Barlow (Barlow, 1961). The two identical networks in this framework, are structured with a backbone encoder followed by three linear layers. Unlike, the contrastive self-supervised frameworks (e.g., MoCoV2), Barlow Twins framework calculates the loss function from the cross-correlation matrix (*C*) computed using the output of two identical networks' representations. When the batch-normalized output of the two branches of the framework are *z*^*A*^ and *z*^*B*^ (see Figure 5B), the loss function can be represented as shown in Equation (2), where *i* and *j* index the vector dimensions of the encoder output. The invariance term ensures robustness from the noise, whereas the redundancy reduction term enforces to make the representation components independent.


(2) $$\begin{array}{l} Loss_{BT}=\underset{\text{invariance term}}{\underset{︸}{\underset{i}{\sum }{\left(1-C_{ii}\right)}^{2}}}+\lambda \underset{\text{redundancy reduction term}}{\underset{︸}{\underset{i}{\sum }\underset{j\neq i}{\sum }C_{ij}^{2}}}\\ \;\text{where}\;C_{ij}=\frac{\sum \left(\overset{A}{\underset{i}{z}}\right)\left(z_{j}^{B}\right)}{\sqrt{\sum {\left(\overset{A}{\underset{i}{z}}\right)}^{2}}\sqrt{\sum {\left(\overset{B}{\underset{j}{z}}\right)}^{2}}} \end{array}$$

### 2.5. Downstream task

We design a learning scheme that converts the classification of *K* classes into *K* binary problems, in this application, *K* = 3. We learn one model for each class and use the image patches belonging to that class. The labeled and object-masked image patches are used to fine-tune the binary classification models to output whether the objects (*Byproduct, Cell*, and *Surface*) are available in the patch or not. These outputs from each classification model merge together to construct the final classification result on a test patch. Therefore, in the final output, one image patch may be assigned more than one class and in some cases, all three classes.

### 2.6. Experiments

We applied the approach described in Figure 1 to automatically classify byproducts, cells, and non-occluded surfaces in the SEM biofilm images. The main objectives of our experiments centered around the following research questions—*Rq1:* Feasibility and effectiveness of automatic classification of byproducts, cells, and non-occluded surface by using state-of-the-art contrastive and non-contrastive self-supervised learning approaches. *Rq2:* Compare and contrast the biofilm image classification performance of MoCo V2 (contrastive self-supervised approach) with Barlow Twins (the non-contrastive self-supervised learning approach). *Rq3:* Evaluate the savings of the expert annotation effort and performance of self-supervised models compared to their fully supervised counterparts. *Rq4:* A qualitative analysis of the classification performance of the self-supervised model through expert feedback.

All the model training and testing tasks were executed on GPU enabled environment using a LAMBDA QUAD Deep Learning Workstation with Intel(R) Core(TM) i9-9920X CPU (3.50 GHz), Nvidia Quadro RTX 6000 GPU with 24 GB memory and 128 GB RAM.

We followed the pre-processing pipeline described in Section 2.2 to prepare the dataset containing 7 grayscale SEM images for model training. Using an image processing tool, we manually cropped out the meta-information that appeared on the images. Next, BSRGAN-based image resolution enhancements were applied on SEM images with 10 or higher micrometers scale whose magnifications were less than 1KX. Then we processed 4X magnification on the images to normalize object sizes across images. Then patches of size 64×64 and 128×128 were generated from each processed, non-annotated image as well as each annotated image for experimentation purposes. The sliding windows technique with a stride size of 2 was used as explained in Section 2.3 to generate these patches. With a patch size of 128 × 128, we obtained 24021 image patches from all 7 images in the dataset. We used the pixel colors and masking process to generate, for each patch, at most three ground-truth labels, one per class. We have used 80% of the dataset to learn representations and 10% of the data to fine-tune the model. Here, we used ten-fold cross-validation to fine-tune strongly annotated data (image-level ground truth annotations). The rest of the 10% data was used as test data to generate the final multi-label classification results.

We implemented MoCoV2 and Barlow twins with ResNet-50 (He et al., 2016) as the encoder architecture using the model configuration recommendations for the best performance as given in (Chen et al., 2020b; Zbontar et al., 2021). We built 3 binary classification networks, one for each class *Byproduct, Cells*, and *Surface*. The prediction from each binary classification model was collectively used to construct multi -label classification result on each input image patch. For example, for the image patch shown in Figure 4, a successful classification model should assign all three classes to the patch. All the experiments were conducted repeatedly with ten random cross-validations to improve the estimated performance and better model generalization.

**Figure 6:**
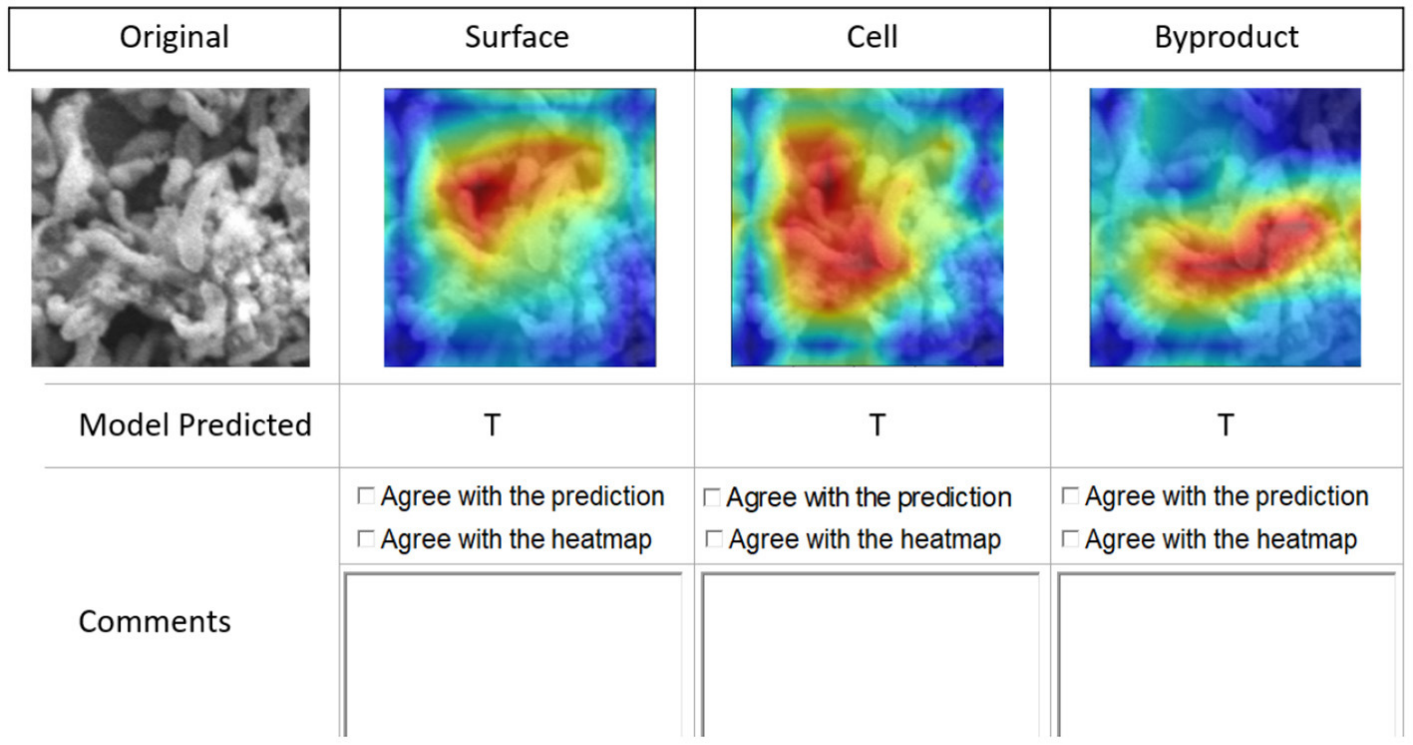
Interface for qualitative feedback. Image in the first row, first column shows an image patch followed by three columns of CAMs showing the true for class *Surface*, true for class *Cells*, and true for class *Byproduct*. Checkboxes and textboxes in the bottom half of the figure are available to input experts' agreements and feedback.

To obtain qualitative feedback, the experts were given 10 patches selected from each image spanning a spectrum of difficulty levels (*easy* to annotate and *hard* to annotate) for manually identifying the three classes. A simple user interface was created (see Figure 6) for experts to provide their qualitative feedback along with comments. The experts inspected each image patch (see Figure 6 first image in the first row) for manual classification in one of the three classes. For each patch, we have provided the original image patch, then three Class Activation Maps (CAM)^1^ (the three images to the right of the image patch in first row in Figure 6) generated by the model for three classes (*Byproduct, Cells*, and *Surface*) (Zhou et al., 2016). Predictions indicate True (T) or False (F), if the model is confident about the presence of the object returns T, else F.

Note that we used CAMs derived from them model outputs about the image classification task to receive feedback from the domain experts beyond model's prediction performance. We used CAMs as a supplementary artifact to visualize and explain model inferences. This allowed the experts to validate whether the model outputs regarding its multi-label classification task were well justified and whether the model's attention when it was predicting a class label(s) was found to be reasonable by experts.

### 2.7. Evaluation metrics

The self-supervised learning models were evaluated with respect to the learned representation quality and fine-tuned downstream performance. These notions are explained below. Initially, we conducted empirical experiments to estimate the best-suited configurations (patch size, batch size, and number of epochs) for learning representations. The representations were learned with all the unlabeled data with different patch sizes (64 × 64 and 128 × 128), different batch sizes (128 and 256), and different epochs (200, 300, and 400). We used all these models with learned representations for further experiment setup.

#### 2.7.1. Linear evaluation for learned representation quality

**Figure 7:**
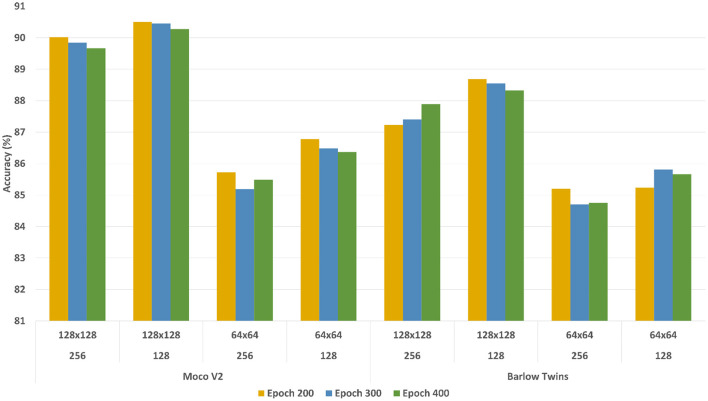
Linear evaluation accuracy comparison of MoCoV2 and Barlow Twins models with different configurations of batch sizes and patch sizes. The first row of the y-axis indicates the patch sizes, the second row indicates batch sizes, and the third row indicates the corresponding self-supervised framework.

To evaluate the learned representations' quality, we conducted a linear evaluation experiment using a linear classification head on the learned representations as discussed in Chen et al. (2020a); Grill et al. (2020); Zbontar et al. (2021). We used standard configurations to run the experiments. During the training stage, only the linear classification head was trained with approximately 10% of image-level ground truth annotations while the encoder models were remained frozen (no weight adjustments for representations). Figure 7 shows the classification accuracy (See Equation 3) with different configurations, where the x-axis represents the classification accuracy and y-axis represents different configurations. The accuracy of the models were calculated based on image-level ground truth annotations.


(3) $$\begin{array}{l} Accuracy=\left(Number\;of\;correct\;predictions\right)/\\ \left(Total\;number\;of\;predictions\right) \end{array}$$

According to the results, both MoCoV2 and Barlow Twins models illustrated the highest classification accuracy with the parameter configuration of patch size 128×128, batch size 128 and trained with 200 epochs. For all the evaluation experiments, at the training time, we used random crop and horizontal flip augmentations, and at test time we used center crop augmentations as suggested in Zbontar et al. (2021). We report the best configuration (patch size 128×128, batch size 128, and trained with 200 epochs) performance across all three classes (*Byproduct, Cell*, and *Surface*) as well as overall averages of the results in Table 1. For comparison purposes, as the baseline, we trained the corresponding three binary classification supervised models (supervised pre-trained ResNet-50 with same encoder architecture used in self-supervised frameworks) (Kornblith et al., 2019) using 100% of the available image-level ground truth annotated data for linear evaluation and 10% of the available image-level ground truth annotated data for fine-tuning evaluation. These models are trained with the same data augmentation as our self-supervised models and standard cross-entropy loss.

**Table 1:** Classification accuracy percentages under linear evaluation on Biofilm dataset.

**Method**	**Accuracy (%)**			
	**Byproduct**	**Cell**	**Surface**	**Overall**
Supervised	**88.32 ±0.74**	**91.04 ±0.51**	**96.21 ±0.03**	**91.85 ±0.42**
MoCoV2	85.73 ± 1.49	89.71 ± 0.80	95.87 ± 0.43	90.44 ± 0.91
Barlow Twins	85.75 ± 1.08	85.10 ± 0.75	95.45 ± 0.26	88.77 ± 0.70

Best results are in **bold**. The results are presented in the format of (*mean*±*std*).

#### 2.7.2. Fine-tuning evaluation

In this experiment setting, we evaluate the data efficiency of each self-supervised model. We fine-tuned each (MoCoV2 and Barlow Twins) model that learned representations for the downstream classification task on the image-level ground truth annotated data for 20 epochs with different portions of the labeled data. However, 10% of the image-level ground truth annotated data showed significantly better classification accuracy. We illustrated the accuracy of the classification tasks from each binary classification model (over each class) as well as averaged overall classification accuracy performance in Table 2. All these results were generated using the best configuration settings (including the patch sizes, batch sizes, and augmentations) mentioned in Section 2.7.1. The accuracy of the models were calculated based on image-level ground truth annotations.

**Table 2:** Classification accuracy percentages under fine-tuning evaluation on Biofilm dataset with all the labeled subset.

**Method**	**Accuracy (%)**			
	**Byproduct**	**Cell**	**Surface**	**Overall**
Supervised	69.01 ± 0.17	62.18 ± 0.28	93.86 ± 0.02	75.01 ± 0.15
MoCoV2	73.52 ± 3.28	73.18 ± 2.36	95.50 ± 0.40	80.73 ± 2.01
Barlow Twins	**78.76 ±3.40**	**74.67 ±2.70**	**96.11 ±0.47**	**83.18 ±2.19**

Best results are in **bold**. The results are presented in the format of (*mean*±*std*).

#### 2.7.3. Qualitative evaluation

The qualitative evaluation of the applicability and performance of the proposed approach for the classification task involved summative and formative assessments by domain experts. The experts assessed the *Sq1:* overall feasibility and the utility of the proposed approach in classifying SEM biofilm images in comparison to manual and semi-automated (ImageJ) classification approaches, and aid in the discovery of class objects, *Fq1:* existence of each class object predicted by the models in the image patches by providing binary (T or F) agreement along with additional remarks and *Fq2:* justification provided by the model for its prediction for the existence of each class in the image patch by providing (T or F) agreement along with additional remarks.

## 3. Results

We report on quantitative analysis results in terms of the evaluation of the robustness of the two self-supervised approaches with respect to parameter variations (*Rq1*) and compared the two self-supervised models along with a supervised model baseline (*Rq2, Rq3*) using linear and fine-tuning evaluations and the amount of annotation savings achieved. In addition to these quantitative analyses results, we also report on the results of our qualitative analyses on the performance of our models by domain experts.

### 3.1. Evaluation of robustness to parameter variations

Figure 7 shows the accuracy on the Y-axis and the parameter settings (patch size and the batch size) on the X-axis. There are three plots in the figure, one plot for each setting of the number of epochs used for model building. From the results, we can observe that the overall performance of both the models trained with the patch size 128×128 significantly outperformed those with patch size 64×64, regardless of the batch sizes and amount of training epochs. The difference in performance is around ~4.5%. One possible reason for this performance difference could be due to the low information content within a 64×64 patch. Hence, we used 128 ×128 as the patch size in all our experiments. Finally, with different training epochs (200, 300, and 400) the models showed almost similar results, hence we preferred 200 epochs during the representation learning stage considering the computational time-saving.

### 3.2. Linear and fine-tuning evaluations on the dataset

In order to evaluate the quality of the representations learned by the self-supervised models in comparison to the supervised baseline (*Rq2)*, a linear classification head was trained while freezing the learned representations. Table 1 summarizes the accuracy and overall performance of the binary linear classification model for each of the three classes. As expected due to the use of 100% image-level ground truth annotations, the baseline supervised model performed better than the self-supervised ones on the linear evaluations. In the linear evaluation, the MoCoV2 model outperformed the Barlow Twins model by approximately by 2%. However, according to the standard deviation (*std*) values, Barlow Twins' results were more consistent than MoCoV2. Although both self-supervised models did not perform as well as the supervised approach in the linear evaluation, their accuracy was pretty close to the supervised model. In terms of the annotation effort (*Rq3*) the two self-supervised models achieved performance comparable to the supervised model baseline while using only 10% of the labeled data.

Similarly, As shown in Table 2, fine-tuned Barlow Twins model shows superior results (83.18%) over both supervised baseline (75.01%) and fine-tuned MoCoV2 (80.73%). Here we report the average accuracy on binary classification tasks as well as overall performance with the same test set used in linear evaluation experiments. These results provide empirical evidence that the Barlow Twins model is more capable of adjusting to perform a downstream task after fine-tuning the weights using limited labeled data when compared to the MoCoV2 method.

### 3.3. Qualitative results

For the summative assessment, *Sq1*, the expert feedbacks were highly positive indicating that the classification performed by the method was useful and applicable to many downstream tasks including identification of image regions with specific class objects, their co-occurrence frequencies to estimate their correlations and other properties of their distribution across the patches. They also found good efficiency improvement in using the proposed approach measured in terms of the time taken to manually analyze the dataset. It was estimated that several orders of magnitude time improvements were obtained by using the proposed approach in comparison to semi-automated approaches based on tools such as ImageJ. This is particularly an impediment to scalability as such manual and semi-automated approaches will become impractical as the number of images increases. Most importantly, the experts compared the class objects discovered by the proposed approach so that they could identify them in raw images and found instances where they agreed with the model prediction and could recognize these objects after reviewing the model predictions.

The domain experts inspected the original image patch, corresponding three image-level annotations (as described in Section 2.3) and the three-class activation maps for evaluating whether (1) The predicted class is present in the original image patch, and (2) The predicted class is present at the location highlighted in the CAM (see Table 3). For *Fq1*, in cases where the domain experts disagree with the machine learning predictions three possible reasons were identified: (1) Annotations were incorrect, (2) Annotations were correct but the class predicted by the machine learning model was incorrect, and (3) Annotations were ambiguous where the domain experts are also uncertain in cases involving overlapping cells and byproducts.

**Table 3:** CAM visualization results.

**Original**	**Byproduct**	**Cell**	**Surface**
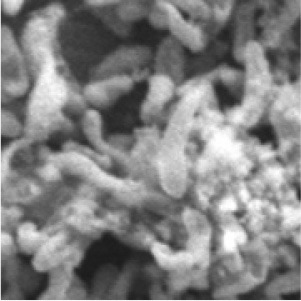	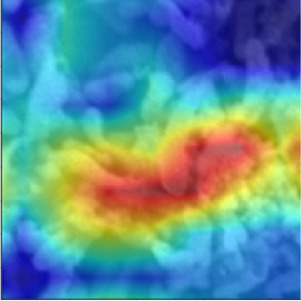	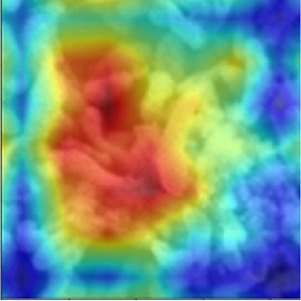	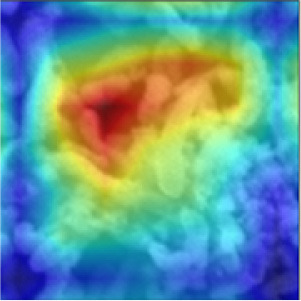
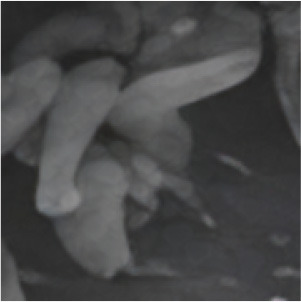	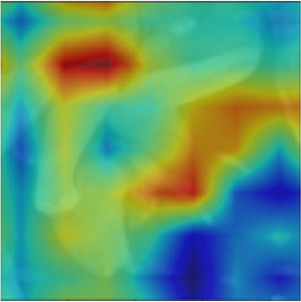	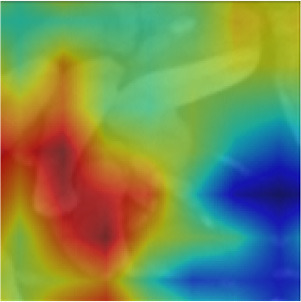	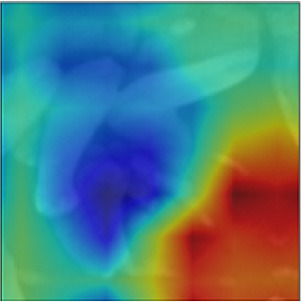
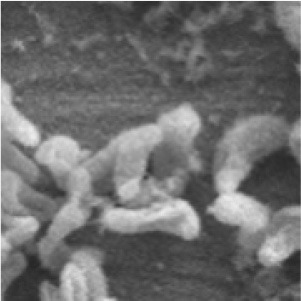	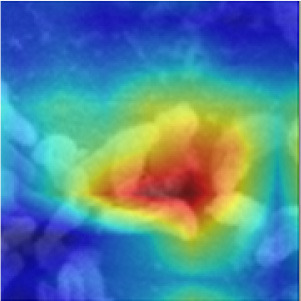	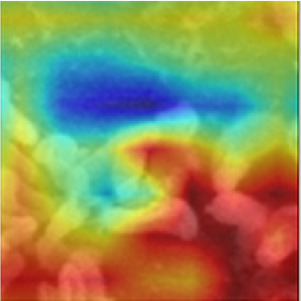	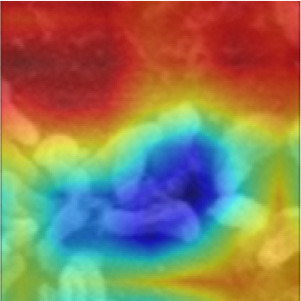
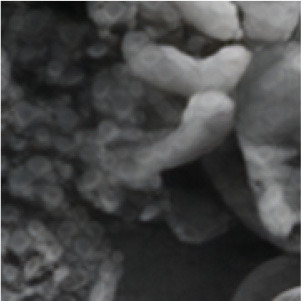	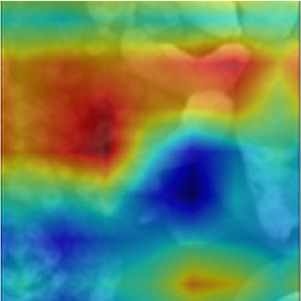	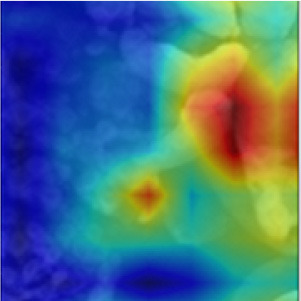	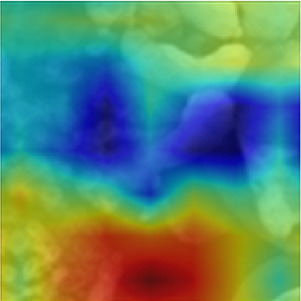

We compare the visualization results obtained with each binary classification model. These visualizations were calculated for the last convolution output of the binary classification models.

In comparison to the *Fq1*, for *Fq2*, the disagreement between the domain experts' assessment and the machine learning model predictions was larger. One of the main reasons for this disagreement was that the present class activation maps scheme could not highlight the presence of a particular class in the image patch when the objects belonging to this class are separated and are present in multiple regions in the image patch. Also, this evaluation metric of the class activation maps is very subjective, particularly on image patches involving overlapping objects belonging to multiple classes. In summary, approximately 98% of the time domain experts' agreed with the model prediction.

## 4. Discussion

Several studies (Jaiswal et al., 2020; Tian et al., 2021; Balestriero and LeCun, 2022) have proposed theoretical analysis to form justifications for the empirical performance of self-supervised approaches. Selection of data augmentation and pretext tasks, dataset biases, and global and local spectral embedding are some of the factors to justify the empirical results. At a more general level, contrastive self-supervised learning frameworks tend to extract better representations of global data's structures whereas non-contrastive approaches are better at grasping local structures in the data. Based on our experiments, we draw the following observations from the experiment results.

Considering the overall classification accuracy results, it is apparent that the proposed self-supervised learning-based pipeline performs significantly better with a smaller amount (~10%) of image-level ground truth annotations. According to the linear evaluation experiments, it is clear that both MoCoV2 as well as Barlow Twins models are capable of retrieving similar quality representations using the unlabeled dataset. However, with the fine-tuning process toward the final classification downstream task using a limited amount of labeled data, Barlow Twins model gained in performance enhancement not only compared to MoCoV2 but also its supervised counterpart. As both the self-supervised models surpassed supervised model performance and considering the qualitative feedback on the model predictions, we can conclude that the representations learned using the unlabeled data lead to better classification accuracy. Moreover, Barlow Twins model is more preferable due to its superior model classification accuracy performance while incurring a substantially lower computational cost compared to MoCoV2. Note that we use pixel-level annotations in contrast to image-level annotations for our classification task. Our earlier work (Bommanapally et al., 2021) based on image level annotation resulted in low-level accuracy and this work addresses this problem by using limited pixel-level annotations.

It is important to discuss the selection of model building for the downstream task. Even though it is apparent that multi-label classification suits the requirement of predicting multiple objects that appear in an image patch, we used the simple and intuitive binary classification models for assigning multiple labels to an image patch. Some of the qualities of the dataset such as imbalance class instance proportions (especially the ratio between *Surface* class and *Byproduct* class was high), and low inter-class variance between classes (*Cell* class and the *Byproduct* class due to sharing similar visual features), binary classification was a favorable choice over direct multi-label approaches such as algorithm adaptation approaches. Even though it is well-known that the adaptation of the binary relevance approach has its potential weakness of ignoring correlations among labels (Zhou and Zhang, 2017), we conjecture that this weakness was mitigated because of the representation learning step of self-supervised learning models which might have captured the correlations.

The effectiveness of the machine learning approaches developed in this paper is shown in classifying the morphologies in SEM biofilm images on metals in which coatings were applied to act as barriers to MIC (Chilkoor et al., 2020) due to sulfate-reducing bacteria. We analyzed biofilm components including cells, cell clusters, and byproducts in different regions (patches) of SEM images of mild steel with graphene coatings. Our classification results provide an automated alternative for experts to analyze different coated areas and provide additional inputs to the domain experts to further control their experimental parameters. Several tools have been employed to extract and evaluate the geometric properties of biofilm microstructures including deep neural network (Buetti-Dinh et al., 2019), BioFilm Analyzer (Bogachev et al., 2018), BiofilmQ (Hartmann et al., 2021), and ImageJ (Rueden et al., 2017). However, these tools characterize the smooth, homogeneous, and non-overlaying geometric structures and are not generic enough to classify the congested biofilm microstructures and byproducts as done in this paper.

Some recent works (Atha and Jahanshahi, 2018; Stoean et al., 2018) have used machine learning approaches to detect corrosion (not MIC) in metals caused by environmental factors. These works employ color spaces to identify discolorations and variations in colors to identify corroded areas in metal surfaces. They use different color channels (RGB, YCbCr, CbCr, and grayscale) and patch sizes (128 × 128, 64 × 64, and 32 × 32) to identify the optimal color spaces to detect corroded metal areas. Supervised deep learning has been used by these methods and image patches are used to increase training data volume similar to our work. Identifying morphologies in SEM biofilm images requires considerable expertise and cannot be done based on the color spaces of SEM images. The intricate nature of these images also makes it difficult to annotate and train supervised deep learning models where a large number of annotated images are needed for training. Given the low throughput of SEM images, our approach similarly deals with a low volume data set applied by patching, but employs self-supervised methods for classification to minimize expert annotation effort. Self-supervised learning provides a promising direction for analyzing SEM biofilm images and can be used in a push-button manner to study and explore biofilm characteristics with minimal expert annotation efforts. According to the experiments in this study, the model performance for grayscale SEM datasets depends on the kernel size of the sliding window technique. Even though a smaller sliding window kernel size (ex, 32 × 32) is useful to achieve an accurate localization, the smaller the input image size, the less number of features a CNN can learn which leads to a decrease in model prediction performance. Given the variations in the shapes and sizes of the cells, cell clusters, non-occluded surfaces, and byproducts our application supports a very large and rich feature space that are better captured by larger size patches.

## 5. Conclusion

In this study, we investigated and presented a self-supervised learning-based pipeline to improve the classification accuracy of MIC constituents from biofilm SEM images using a limited volume of annotated data. Even though the data generation has grown on an exponential scale, manually annotating the data remains a challenging concern as it is expensive and needs deep domain knowledge. The SEM dataset used in this study can be recognized as not only challenging to annotate but also to generate in large volumes. We have experimented with several aspects of image data pre-processing, self-supervised learning and evaluated the performance on the classification tasks, both quantitative and qualitative, by juxtaposing two self-supervised learning frameworks, MoCov2 (contrastive) and Barlow Twins (non-contrastive) on the dataset. Our study clearly showed that self-supervised model (Barlow Twins) built using our pipeline was able to produce 83.18% classification performance on the SEM image patches with the patch size of 128 × 128 while reducing the requirement of labeled data by at least 90%. Further, the qualitative evaluation showed over 98% agreement on summative and formative assessments by domain experts with the model predictions. Although the binary classification models used for multi-label classification might have the weakness of ignoring correlations among labels during the downstream classification, we believe that most of the correlations were captured during the representation learning stage. Given the potential of state-of-the-art self-supervised learning to mitigate the requirement of the large volume of manual label annotations, we plan to extend this work to perform object segmentation tasks and to further extract quantitative measures of the objects such as area and length. We also plan to extend the experiment setup with specific biofilm image augmentations, and additional self-supervising methods.

## Data availability statement

The data analyzed in this study is subject to the following licenses/restrictions: to access the dataset, please request. Requests to access these datasets should be directed to VG, venkataramana.gadhamshetty@sdsmt.edu.

## Author contributions

DA, MM, MA, and MS contributed to conception and design of the study and wrote the paper. DA and MA implemented the pipeline of models and performed the experiments. PC, JK, and VG reviewed the paper. JK and VG organized the database. PC contributed to funding acquisition. All authors contributed to manuscript revision, read, and approved the submitted version.

## Funding

This project was partially supported by NSF EPSCoR RII Track 2 FEC #1920954.

## Conflict of interest

The authors declare that the research was conducted in the absence of any commercial or financial relationships that could be construed as a potential conflict of interest.

## Publisher's note

All claims expressed in this article are solely those of the authors and do not necessarily represent those of their affiliated organizations, or those of the publisher, the editors and the reviewers. Any product that may be evaluated in this article, or claim that may be made by its manufacturer, is not guaranteed or endorsed by the publisher.

## Footnotes

1 The heated (red) regions in a CAM show the location of the patch where the model has high confidence about the presence of that particular class label.
